# Modern Sociology

**Published:** 1901-05-04

**Authors:** 


### Modern Sociology.

### THE TROUBLES OF A MATCH-MAKER.

The match-maker in question is not the scheming mother of fiction, but the man who makes those phosphorus-tipped splinters of wood wherewith we light our gas, our fires, our tobacco. His troubles are concentrated in that phosphorus tip. Two kinds of phosphorus are used in the making of matches, yellow—it is white originally, but becomes yellow on exposure to the air—and red. The yellow phosphorus emits fumes which are dangerous to those who work with it; the red is perfectly harmless. The yellow is used for the manufacture of ordinary matches; the red for that of the safety matches which light only on the box. In the interests of the workers it might be desirable that only safety matches should be used, and this is made compulsory by law in Denmark, and has been resolved upon in Switzerland; while in Russia a tax is imposed upon the manufacture of yellow phosphorus matches, which is having the effect of displacing them in favour of the safety kind. But the yellow phosphorus match continues to hold its own, especially in countries which manufacture for export, chiefly because of its convenience, but partly also because it has been found, especially in hot and humid climates, that the common match keeps better and resists damp more successfully than the safety one.

The use of yellow phosphorus is liable to produce among the workers exposed to its action in mixing the phosphorus paste, dipping the matches, drying and boxing them necrosis of the teeth and jaws, an ailment whose name the workers contract into "phossy jaw." Besides this direct and obvious action of phosphorus, it is said that in time the bones of workers in match factories become extremely brittle, and break very easily, even where no known accident has befallen them. This observation was first made by Dr. Magitot, of Paris, who also found out that many of the patients in Paris hospitals who were suffering from pulmonary consumption were match-makers; while in consequence of breathing the irritating fumes arising from handling the matches, and the frequent fires that arose during the boxing of them, bronchitis was common among the workers. This, however, was as long ago as 1856, when the ventilation and general sanitary arrangements of factories were much less good than is the case at present.

The chief trouble is, however, "phossy jaw." While admitting the seriousness of this disease and the importance of taking every precaution to prevent it, it should in justice be said that the facts and figures which have been collected by the Government investigators—Professor T. E. Thorpe, principal chemist of the Government Laboratory; Professor Thomas Oliver, and Dr. George Cunningham, senior dental surgeon to the London Hospital—do not bear out the sensational statements on the subject that sometimes find their way into the press. A great deal depends on the teeth, and Dr. Oliver states that "experience has shown that people with sound, healthy teeth can be exposed to these (phosphorus) fumes, and no harm arises unless the constitution is by nature scrofulous or tubercular, or has become enfeebled by intemperate use of alcohol." Unfortunately, many of the workers in our match factories are ill-nourished, anæmic creatures, who would be a sure prey to any ailment to which their work exposed them. Yet, even so, the statistics of "phossy jaw" are not so alarming as is sometimes made out. In 23 factories visited the number of workers was 3,134 persons, of whom 1,613, or rather more than half, were engaged in non-phosphorus processes. During the five years 1894 to 1898 there have been recorded 36 cases of phosphorus necrosis in the United Kingdom. Three of those cases are known to have ended fatally. All these were men engaged in dipping matches in the phosphorus paste. Twenty women were among the sufferers, but all seem to have recovered. About 80 per cent. of the cases of phosphorus necrosis recover, though there is often considerable disfigurement.

In these statistics the employés of one factory—the Diamond Company's works at Liverpool—are not included, as the bulk of the work there is done by machinery, and the dangers of working with phosphorus are minimised. The figures therefore do not present the facts in an unduly favourable light. By the machine there employed, the block of wood is placed in the machine, and by mechanical means split into slips the size of a match, dipped first in paraffin and then in phosphorus paste, dried by passing over a series of drums, and boxed without touch of human hands. At the end of the machine sit one or two girls, who receive the boxes, add one or two matches if it is not quite full, and slip on the outer covering. They and the men who mix the phosphorus paste are the only persons in any way exposed, and there has been no case of necrosis in this factory.

With regard to the mixing, which cannot be done without the interposition of human intelligence, Dr. Thorpe recommends that it should be done in closed vessels, and also speaks favourably of a suggestion by Mr. A. Newlands, one of H.M. Inspectors of Factories, that a current of air impregnated with oil of turpentine should be allowed to pass over the tub when either mixing or dipping is being carried on. It has been found that the presence of phosphorus in the air prevents the phosphorus from fuming. As it is through decayed teeth that the phosphorus finds its way into the system, it is also desirable that the teeth of the workers should be examined periodically. This is done voluntarily by some factories in this country, but in several continental countries a quarterly inspection is made compulsory by law, and some manufacturers insist on their own account on their employés submitting to a more frequent examination. Good teeth being so important, it happens that this is a trade which it is safer for young persons to follow than their seniors, though, on the other hand, they are likely to be more careless as to its dangers. Some firms provide gargles of permanganate of potash for their workers, and washing conveniences are always provided. Of course this is not always efficacious unless there is a reliable superintendent to make sure that the workers avail themselves of these advantages.

But with all possible precautions, there will always be the risk of cases of "phossy jaw" until the ideal match is invented—the match destitute of yellow phosphorus which will yet strike anywhere. The Belgian Government has offered a prize of £2,000 to whoever shall produce it. Some French chemists claim to have succeeded in the feat. They use a sesquisulphide of phosphorus, but as this is said to be a rather unstable body time will be required to prove that these matches will keep their combustible properties for a lengthened period, and in any climate. But the interest that has been aroused in the subject will lead chemists to continue making experiments.

